# Supplementary material for: State modulation in spatial networks with three interneuron subtypes
Source: Sci Adv. 2025 Jun 25;11(26):eads9134. doi: 10.1126/sciadv.ads9134 (PMC13108820; doi:10.1126/sciadv.ads9134)
Supplement: Supplementary file 1 — Figs. S1 to S17 Legends for movies S1 to S3 References [file sciadv.ads9134_sm.pdf]

Science Advances
AAAS

Supplementary Materials for

State modulation in spatial networks with three interneuron subtypes

Madeline M. Parker *et al.*

Corresponding author: Chengcheng Huang, huangc@pitt.edu

Sci. Adv. **11**, eads9134 (2025)
DOI: 10.1126/sciadv.ads9134

The PDF file includes:

Figs. S1 to S17
Legends for movies S1 to S3
References

Other Supplementary Material for this manuscript includes the following:

Movies S1 to S3

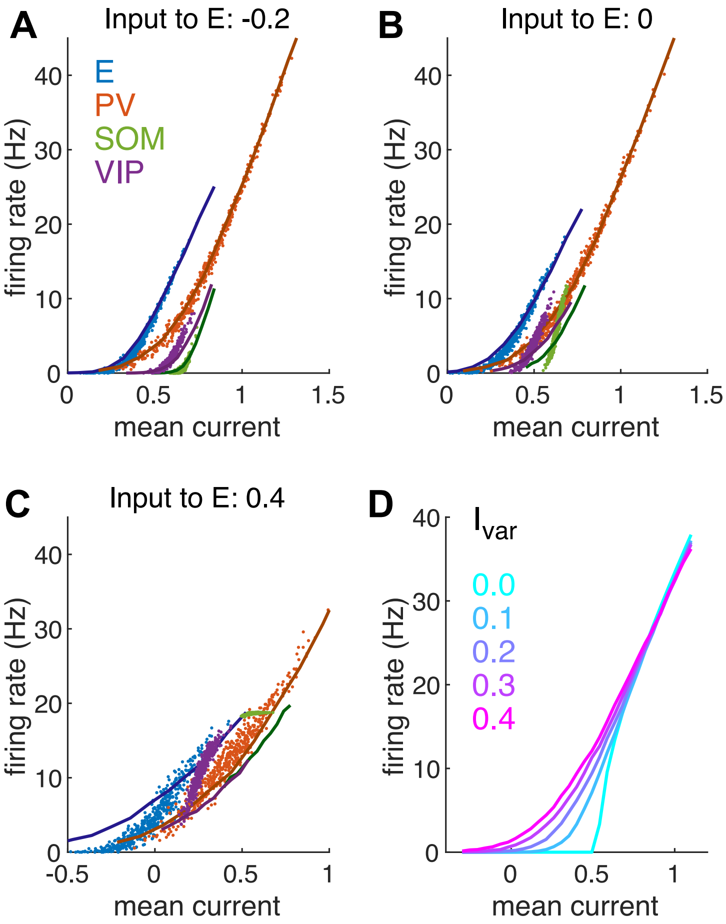

Figure S1: Rate-current transfer functions of each neuron population in the Subcircuit Asynchronous (A), Weakly Synchronous (B) or the Strongly Synchronous (C) state. Each dot represents the mean total current and firing rate of one neuron. There were 500 neurons sampled from each neuron population. Solid curves are the rate-current transfer functions using the population-averaged current variance measured in network simulations of different states and the EIF neuron model parameters for each population. The rate-current transfer functions were calculated by simulating an EIF neuron model (Equation 1 in Methods) with input ($I(t)$) modeled as an Ornstein–Uhlenbeck process of two time scales: $\tau_r dI = (-I + x)dt,\ \tau_d dx = -xdt + JdW$, where $\tau_r = 1$ ms and $\tau_d = 5$ ms were chosen to be the same as those of the excitatory synapses. $J = \sqrt{I_{\text{var}}(\tau_d + \tau_r)}$ such that the variance of $I(t)$ is I_{var}, where I_{var} was the population-averaged current variance measured in network simulations. (D) The rate-current transfer function of an EIF neuron with different current variance I_{var} (using the E neuron parameters). Note the different x-axis range in C from that in A-B.

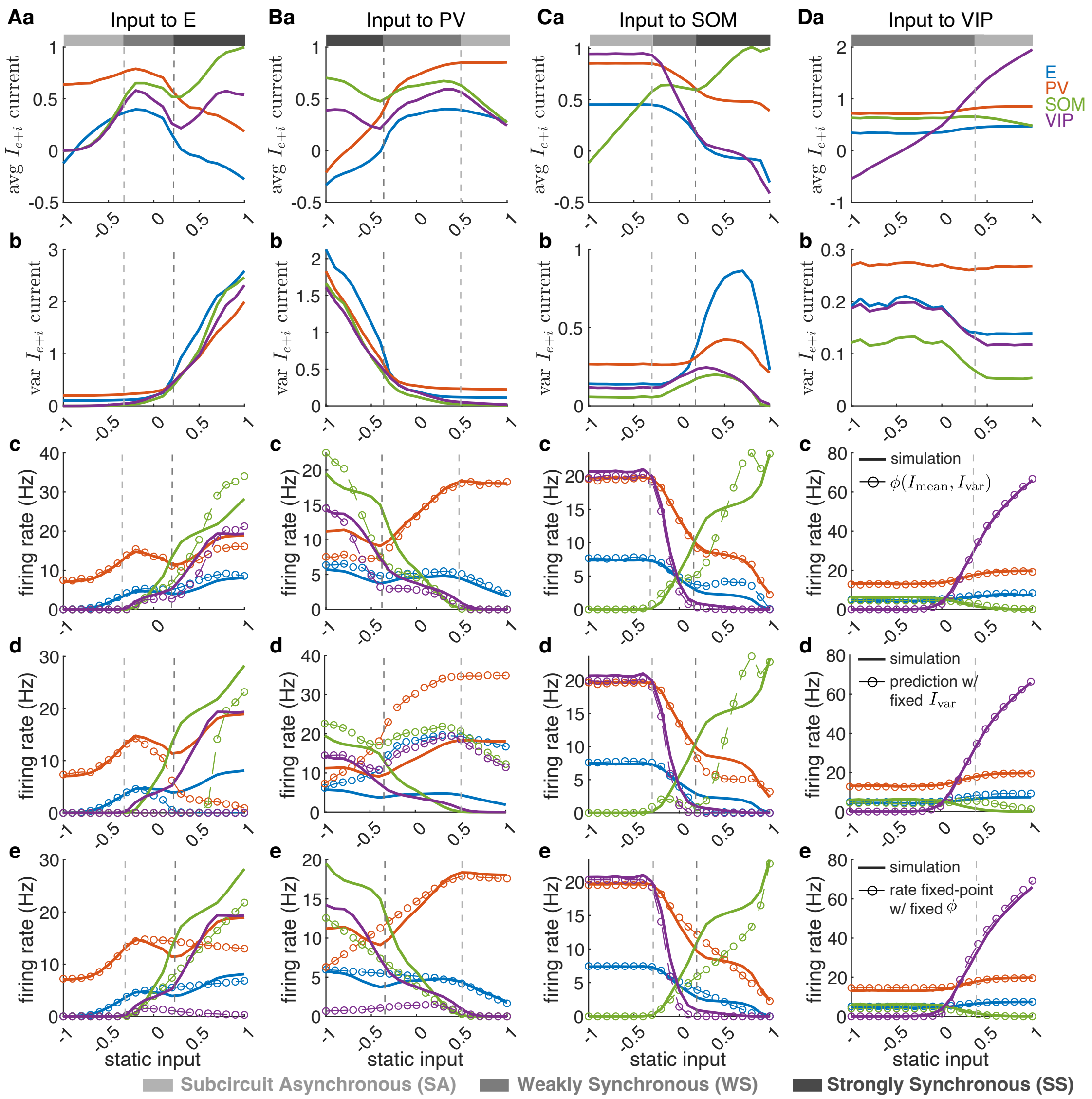

Figure S2: **Changes in the mean and variance of synaptic currents and predictions of firing rates as external input is applied to each population. Related to Figure 3.** Static input is applied to the E (A), PV (B), SOM (C) or VIP (D) population. Row (a): Average total synaptic current to each population. Row (b): Population-averaged variance of the total synaptic current to each population. (c) Dashed curves with circles are predictions of firing rates based on rate-current transfer functions, $\phi(I_{\rm mean}, I_{\rm var})$, where $I_{\rm var}$ was the population-averaged current variance measured in network simulations (as shown in (b)). The population firing rate was predicted as the transfer function averaged over the distribution of mean currents ($p(I_{\rm mean})$) from each cell population, i.e. $\int \phi(I_{\rm mean}, I_{\rm var}) p(I_{\rm mean}) dI_{\rm mean}$. Solid curves are population firing rates from network simulations, same as in Figure 3Aa-Da. (d) Dashed with circles: predictions of firing rates, $\phi(I_{\rm mean}, I_{\rm var})$, where $I_{\rm var}$ was fixed at the value measured when input = -1 for each population. Solid: same as in (c). (e) Dashed with circles: the solution of the rate equations $r_\alpha = \phi(\sum w_{\alpha\beta} r_\beta + w_{\alpha X} r_X + \mu_\alpha)$, $\alpha \in \{e, p, s, v\}$, where $w_{\alpha\beta} = J_{\alpha\beta} \bar{p}_{\alpha\beta} N_\beta$ is the effective weight and $r_X = 0.01$ kHz. The transfer function $\phi(u)$ was fixed to be those with $I_{\rm var}$ from the asynchronous state (input to E = -0.2 case; Figure S1A). Solid: same as in (c).

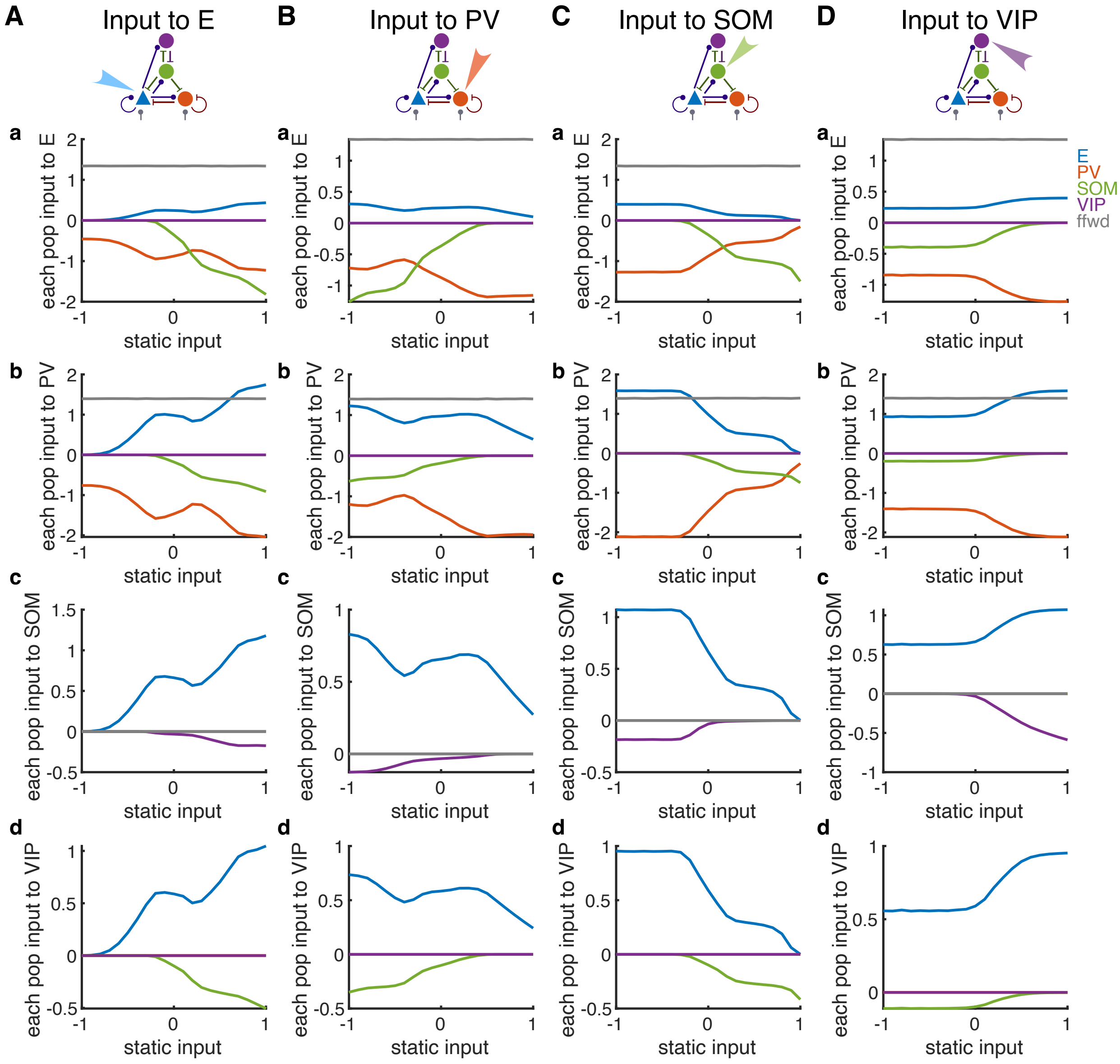

Figure S3: **Average synaptic currents from different population sources across modulation states. Related to Figure 3.** Static input is applied to the E (A), PV (B), SOM (C) or VIP (D) population. Row (a): average input to the E neurons from E (blue), PV (red), SOM (green), VIP (purple) neurons or neurons in the feedforward layer (grey). The static input was not included. Row (b): same as (a) for inputs to PV neurons. Row (c): same as (a) for inputs to SOM neurons. Row (d): same as (a) for inputs to VIP neurons. The average input from population β is $J_{\alpha\beta}\bar{p}_{\alpha\beta}N_\beta r_\beta$, which is proportional to the rate of population β. Therefore, inputs from the same population (curves of the same color across rows) have the same shape but with different coefficients.

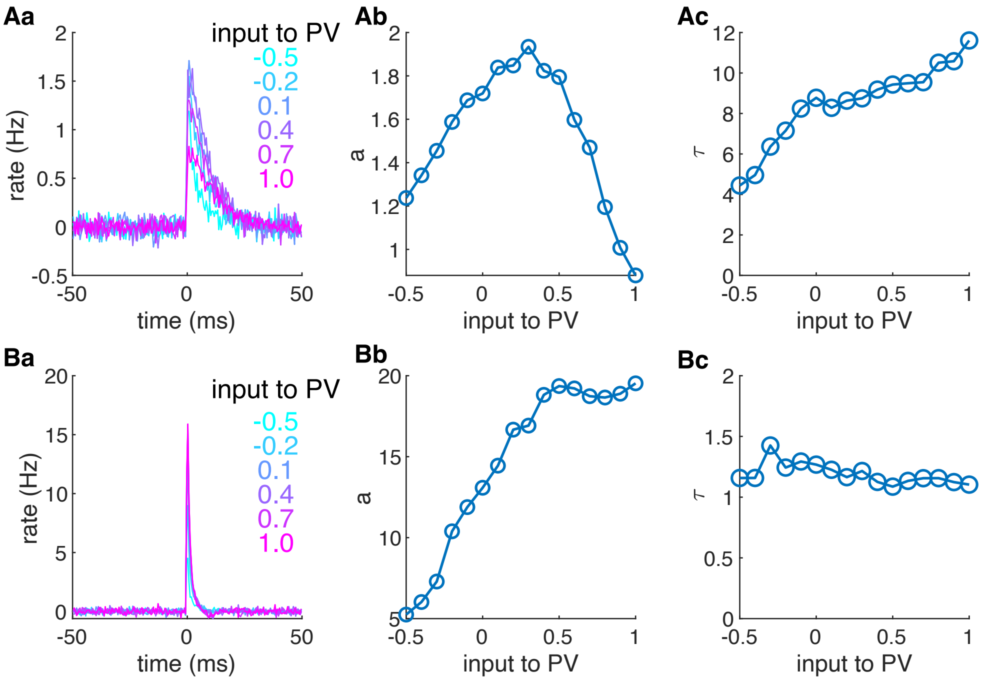

Figure S4: **Linear response kernels of the E (A) and the PV (B) neurons with various levels of input applied to the PV neurons.** (a): The average firing rate response of individual uncoupled E or PV neurons to an impulse input current at time zero ($0.5\delta(t)$), which is denoted as linear response kernels ($h(t)$). The neurons receive colored noise, $I(t)$, modeled as an Ornstein–Uhlenbeck (OU) process of two time scales: $\tau_r dI = (-I + x)dt,\ \tau_d dx = -xdt + JdW$, same as in Figure S1A-C. $\tau_r = 1$ ms and $\tau_d = 5$ ms were chosen to be the same as those of the excitatory synapses. The mean of the OU noise matched the distribution of mean total current over each neuron population and the variance of the noise matched the population-averaged current variance measured in network simulations for each external input case. J was set to $J = \sqrt{I_{\text{var}}(\tau_d + \tau_r)}$ such that the variance of $I(t)$ was I_{var}. Each linear response kernel in (a) was fitted with an exponential function, $ae^{-t/\tau}$, $(t > 0)$. The fitted parameters, a and τ, are shown in (b) and (c), respectively, for different external input values to PV. We find that the response gain (a) of PV neurons increases by several folds as input to PV neurons increases (Bb), which means that small fluctuations in the input current to PV result in larger response in PV population rate when PV neurons receive more external inputs. Meanwhile, the response gain of the E neurons first increases and then decreases when the network is about to transition to the asynchronous state (Ab). The reduction in the response gain of the E neurons also contributes to the desynchonization of network dynamics.

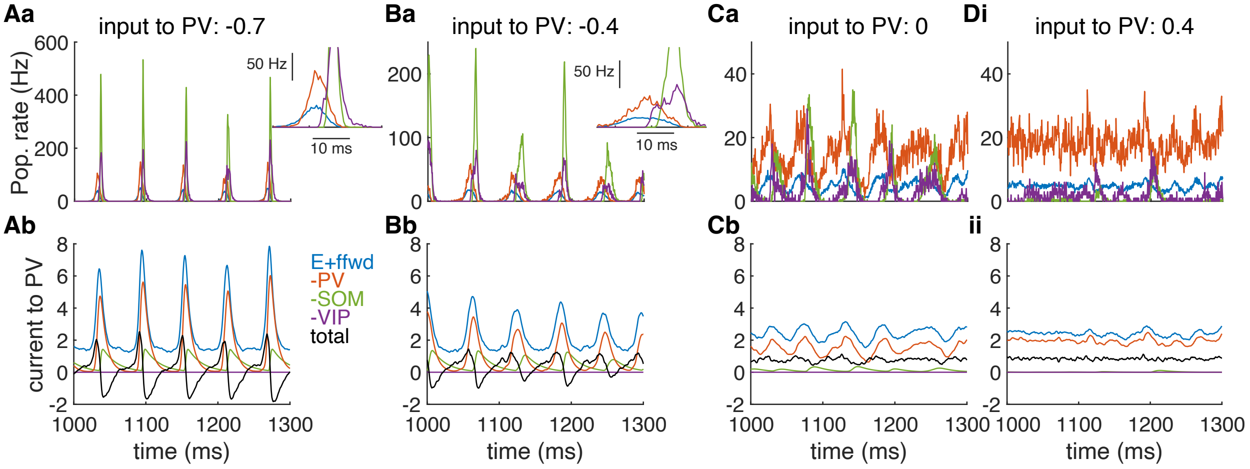

Figure S5: **Example traces of firing rates and input currents in different dynamical states.** The static inputs applied to PV neurons are -0.7 (A), -0.4 (B), 0 (C) or 0.4 (D). Row (i): Population-average rates of E (blue), PV (red), SOM (green) and VIP (purple) neurons. Note that the y-axis has different ranges in Aa-Da. Insets in Aa and Ba: Zoomed-in view of the population rates during one oscillation cycle. Row (b): Population-averaged input currents from different sources to PV neurons. Blue: excitatory currents from E neurons and feedforward projecting neurons. Red: absolute value of the inhibitory current from PV neurons. Green: absolute value of the inhibitory current from SOM neurons. Purple: absolute value of the inhibitory current from VIP neurons. Black: total input current. When the input to PV neurons is very negative, the total current to PV neurons remains at a low baseline at the end of an oscillation cycle (Ab, black), which makes the PV neurons lag behind E neurons slightly at the beginning of the next cycle (Aa, inset). A higher input value to PV neurons increases the baseline current level for PV neurons at the end of each cycle, which allows their firing rate to rise in-phase or earlier than the E neurons at the beginning of each cycle (Ba, inset). The earlier firing of PV neurons suppresses the peak of E firing rate and makes the spiking of E neurons more spread out within a cycle. The spread of E spiking reduces the coherence level and reduces the recruitment of SOM neuron activity. Importantly, an increase in PV firing rate also suppresses PV neurons themselves, because of the strong coupling within the PV population. Therefore, PV neurons tend to fire in tandem with E neurons and they do not exert excessive inhibition like the SOM neurons.

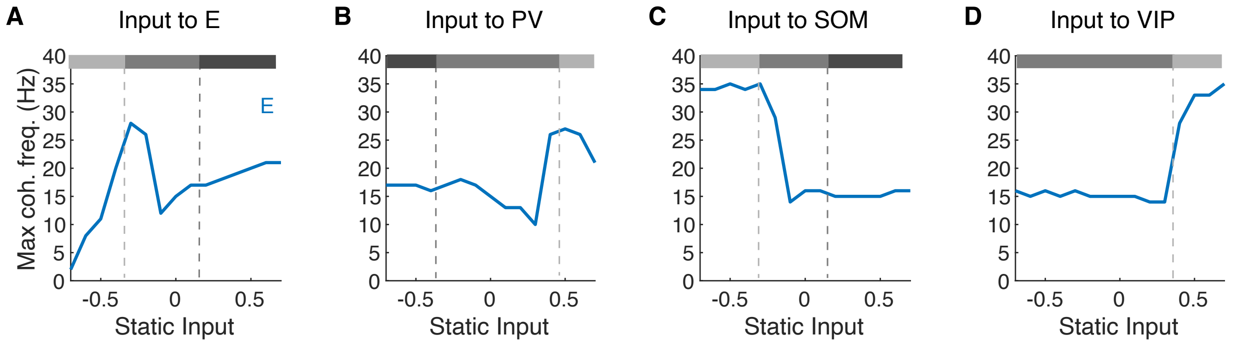

Figure S6: **The frequency of maximum coherence of the E neurons across modulation input values. Related to Figure 3.** Input is applied to E (A), PV (B), SOM (C) or VIP (D) neurons. Grey-scale bars above each plot represent network activity state at the corresponding input value (Subcircuit Asynchronous: light grey; Weakly Synchronous: moderate grey; Strongly Synchronous: dark grey).

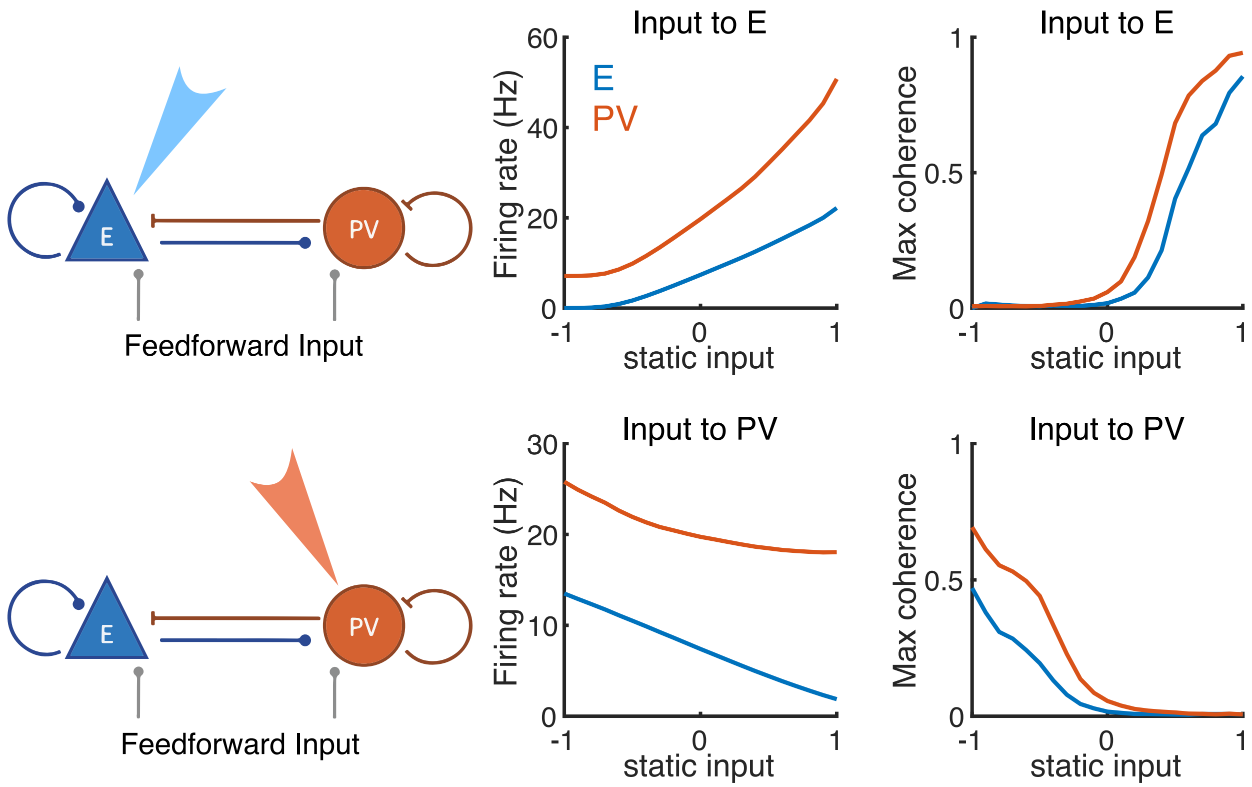

Figure S7: **Modulations of firing rates and maximum coherence in the E-PV subcircuit. Related to Figure 4.** Static external input was targeted to E (top row) or PV (bottom row) neurons. Firing rates and network synchrony are tethered to change in the same direction in the E-PV subcircuit. That is, stimulating E neurons increases the firing rates and coherence of both E and PV neurons, while stimulating PV neurons decreases firing rates and coherence in both populations. The paradoxical effect where stimulating PV leads to a reduction in PV firing rate suggests that the E-PV subcircuit is in the inhibition-stabilized regime (*75–77*). Network parameters were the same as those Figures 1-4 except that we removed SOM and VIP populations.

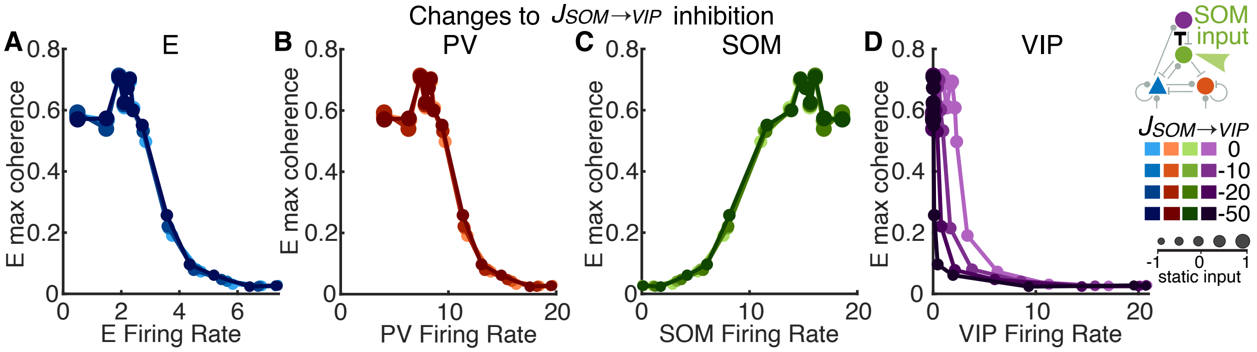

Figure S8: **SOM→VIP connection strength has little effect on modulation patterns. Related to Figure 5.** Static input was applied to SOM neurons.(A) E, (B) PV, (C) SOM, and (D) VIP population rates compared to E maximum coherence in networks with different SOM→VIP connection strengths, $J_{SOM \to VIP}$. Only the relation to VIP firing rate (panel D) is different in networks with different $J_{SOM \to VIP}$. In networks with larger $J_{SOM \to VIP}$ inhibition, SOM is able to suppress VIP at a lower rate, resulting in the darkened curves shifting leftward (D). Therefore, altering the connection strength of $J_{SOM \to VIP}$ exclusively affects the VIP population and does not influence how the rest of the network responds to external input. Note that $J_{SOM \to VIP} = -10$ is the default circuit parameter used in the main text.

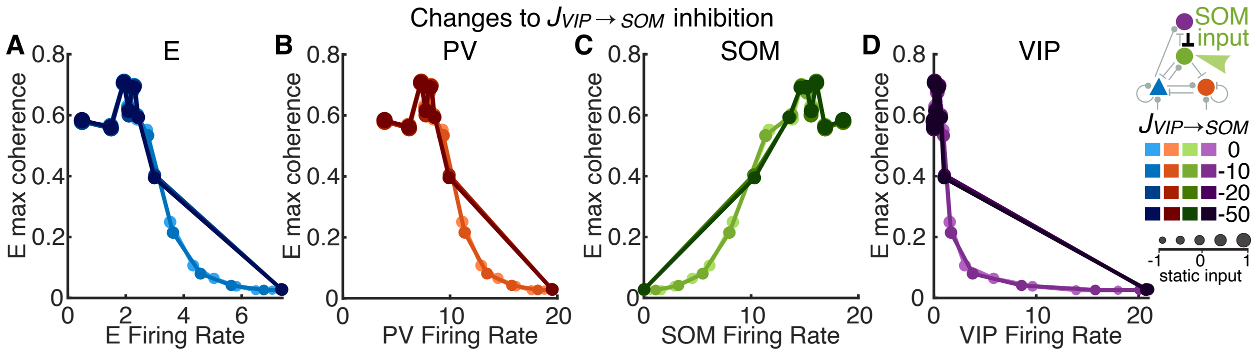

Figure S9: **VIP→SOM connection strength has little effect on modulation patterns. Related to Figures 5, 6.** Same format as fig. S8. Static input was applied to SOM neurons. Modulation patterns are the same across VIP→SOM connection strengths $J_{VIP \to SOM}$ except for in the network with the largest strength (darkest color). Networks with large $J_{VIP \to SOM}$ become sensitive to small changes in external input to SOM. The firing rate of SOM neurons switches from zero to above 10 Hz and the rate of VIP neurons switches from about 20 Hz to near zero as input to SOM increases slightly (one step in panels C,D). Therefore, networks with large inhibition from VIP to SOM exhibit the WS state over only a limited parameter range and switch relatively abruptly between the SA and the SS states as input varies. Note that $J_{VIP \to SOM} = -10$ is the default circuit parameter used in the main text.

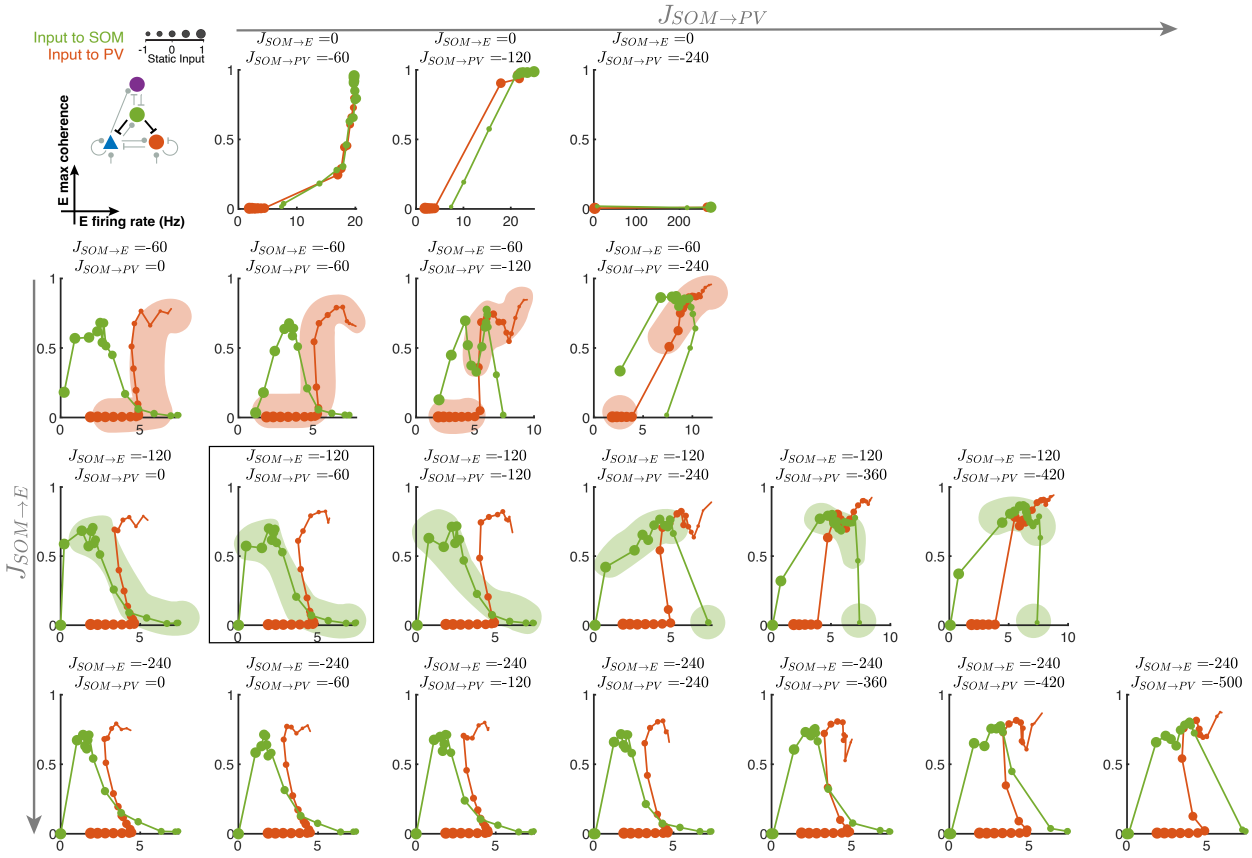

Figure S10: **Modulation patterns in networks with different SOM→E and SOM→PV connection strengths. Related to Figure 5.** Static input was either applied to PV neurons (red) or SOM neurons (green). Each plot represents the maximum coherence of E neurons versus the average firing rate of E neurons. Marker sizes correspond to increasing static input to the target population. Rows represent (negative) increases in synaptic connection strength of SOM→E. Columns represent (negative) increases in synaptic connection strength of SOM→PV. The boxed plot features the same parameters as the default network. We find that when $|J_{SOM\to E}| > |J_{SOM\to PV}|$ (lower triangle of the plots), the shapes of the modulation patterns for both input cases remain qualitatively consistent. When $|J_{SOM\to PV}|$ is much larger than $|J_{SOM\to E}|$, the network exhibits abrupt changes from the SA to the SS state, and the firing rate and coherence of E neurons tend to vary in the same direction over all levels of input to PV. The orange shading in row two and green shading in row three highlight examples of changes in modulation patterns as $|J_{SOM\to PV}|$ increases (across columns in the same row). Discontinuities in the shading are abrupt changes between adjacent dots (i.e., small changes in external input leading to large changes in coherence) along the modulation path.

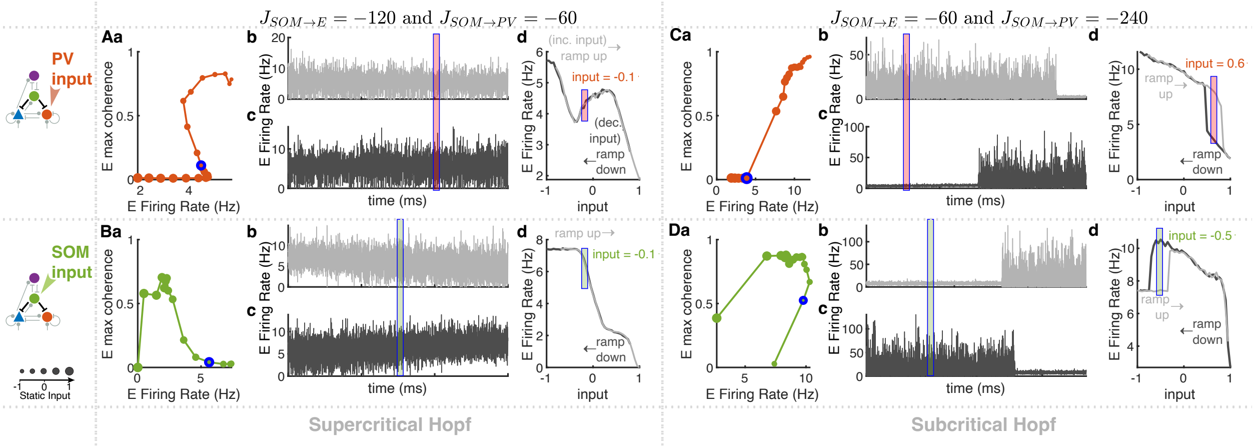

Figure S11: **Hysteresis effects in networks with strong SOM→PV inhibition in response to ramping input. Related to Figures 3,4,5.** (A,B) The default network ($J_{SOM\to E} = -120$ and $J_{SOM\to PV} = -60$; Figures 3,4) with input applied to PV (A) or SOM (B) neurons. (C,D) Same as A,B for a network with stronger SOM to PV inhibition ($J_{SOM\to E} = -60$, $J_{SOM\to PV} = -240$) with input applied to PV (C) or SOM (D) neurons. Panel (a): Modulation path of E population firing rates versus E maximum coherence with varying static input (same format as Figures 4A, S10). The blue outlined marker in (a) represents the input value that is indicated in panels (b)-(d) by colored rectangles. Note that each panel (a) was generated with a sequence of fixed values of static input to the indicated population, and not with ramping input, which changes in time. Panel (b): The population-averaged firing rate of E neurons as a function of time with slowly increasing input (*ramp up* case). Panel (c): same as panel (b) for slowly decreasing input (*ramp down* case). In the ramping input cases, external inputs were increased or decreased by a small incremental change, ±0.05, every 5 seconds. The 5-second interval allows sufficient time for the network to converge to a stationary state at the given input value. The colored rectangle in panels (b) and (c) indicates time intervals of the same input value in both ramping cases, which are aligned in time for comparison. Panel (d): E population-average firing rates calculated within each 5-second interval of ramping input. The initial 250 ms of each interval was excluded to avoid transient activity. In the default network with $|J_{SOM\to E}| > |J_{SOM\to PV}|$ (A,B), the E firing rate is the same for each fixed input value in both *ramp up* and *ramp down* cases. This suggests that there is no co-existence of multiple network states for any input value and that the transition from the SA to the SS state is likely through a supercritical Hopf bifurcation where the amplitude of oscillation increases gradually after bifurcation. In contrast, in a network with $|J_{SOM\to E}| < |J_{SOM\to PV}|$ (C,D), the same input value results in different dynamic states in the *ramp up* and *ramp down* cases (Cb-Cd,Db-Dd, regions indicated by colored rectangles). This hysteresis effect demonstrates the co-existence of two network solutions, one asynchronous and one synchronous oscillation, over a range of input values. This suggests that oscillations arise via a subcritical Hopf bifurcation, where there is a sudden jump in the amplitude of oscillations after the bifurcation point, in networks with strong SOM→PV inhibition.

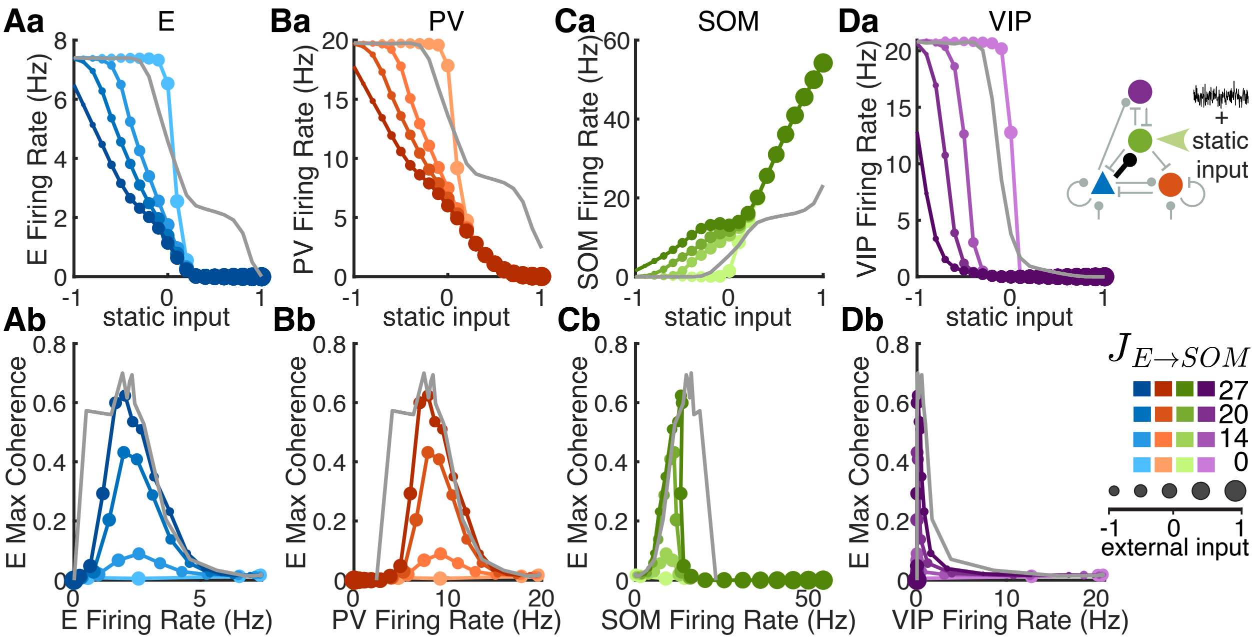

Figure S12: **Population rates and coherence in networks with different E→SOM connection strengths. Related to Figure 6.** Both static input and colored noise were applied to SOM neurons. The colored noise was constructed as an OU process to match the mean and variance of the recurrent excitatory input that SOM neurons receive in the default network without external input (same noise input as in Figure 6A,C). Static input varied from -1 to 1. Column: (A) E, (B) PV, (C) SOM, (D) VIP population. Row (a): Average firing rates of each cell population with respect to static input value. Row (b): The maximum coherence of E neurons compared to the population firing rates of each cell type. The grey curves are from the default network ($J_{E\to SOM}$ = 27), with SOM neurons receiving static input without the OU noise.

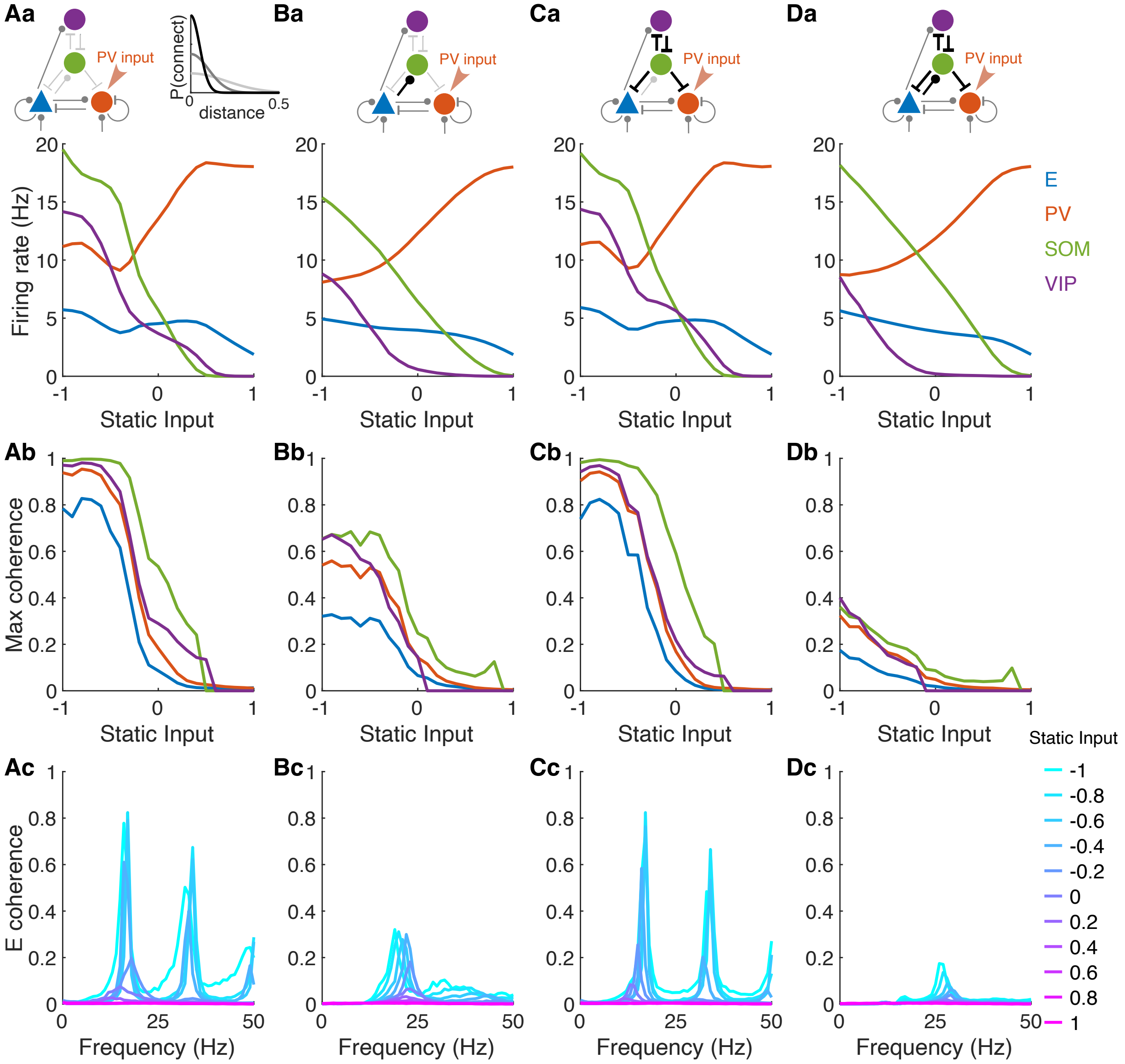

Figure S13: **Impacts of the projection widths of SOM connections on network synchrony. Related to Figure 7A.** Networks in A-D are the same as those in Figure 7Ai-iv, respectively. Row (a): Average population firing rates of each population as a function of static input value. Row (b): Maximum coherence in each population as a function of static input value (same as Figure 7A). Row (c): E population coherence as a function of frequency for several static input values. (A), default network where connections to and from SOM neurons were broader (width = 0.2 mm) compared to other connections (width = 0.1 mm). (B), E→SOM connection width is narrowed to 0.05 mm while keeping all other connection widths the same as those in the default network. (C), SOM connections other than E→SOM, i.e. VIP→SOM, SOM→E, SOM→PV and SOM→VIP connections, are narrowed to 0.05 mm while keeping all other connection widths the same as those in the default network. (D), all connections from and to SOM neurons are narrowed to 0.05 mm, while keeping all other connection widths the same as those in the default network. In all four networks, static input was applied to the PV neurons and varied from -1 to 1.

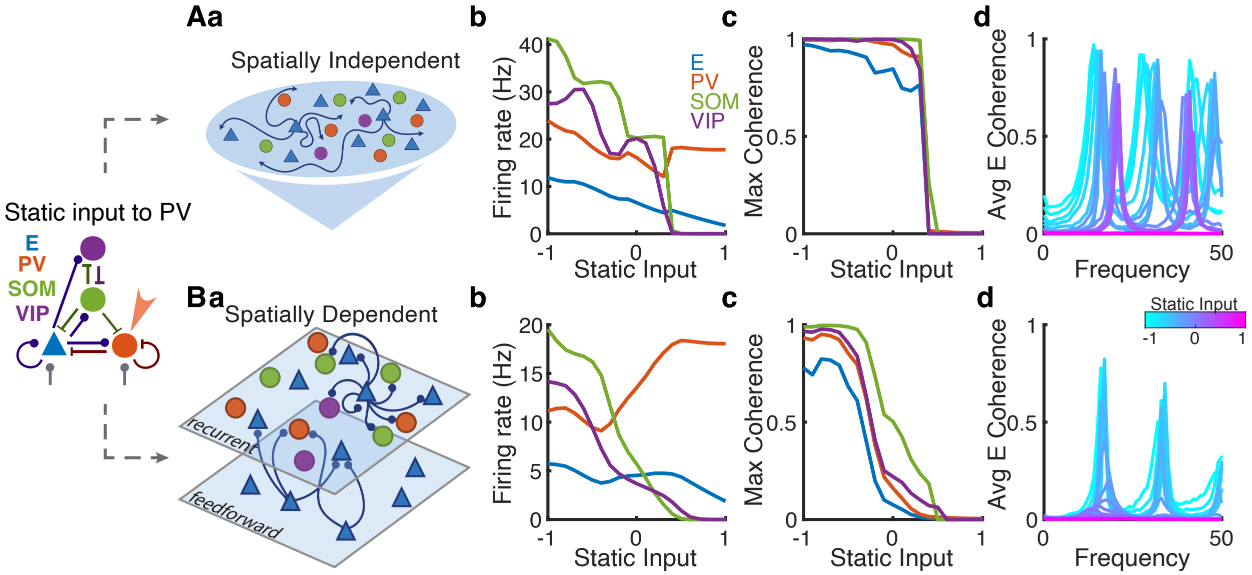

Figure S14: **Sharp transitions in networks with no spatial structure. Related to Figures 3, 7.** Static input was applied to PV neurons. (Aa-Ad) Firing rate and coherence in the four neuron populations in networks with no spatial structure, meaning that the connection probability between two neurons does not depend on distance. A sharp transition from the asynchronous to the strongly synchronous state occurs as input is increased. (Ba-Bd) The same quantities for networks with spatial structure, as also shown in Figure 3B. The spatially dependent network exhibits a more gradual transition and the existence of weakly synchronous state over a range of input values. The parameters of the networks in A and B are the same except for the connection widths, $\sigma_{\alpha\beta}$ (Table 1).

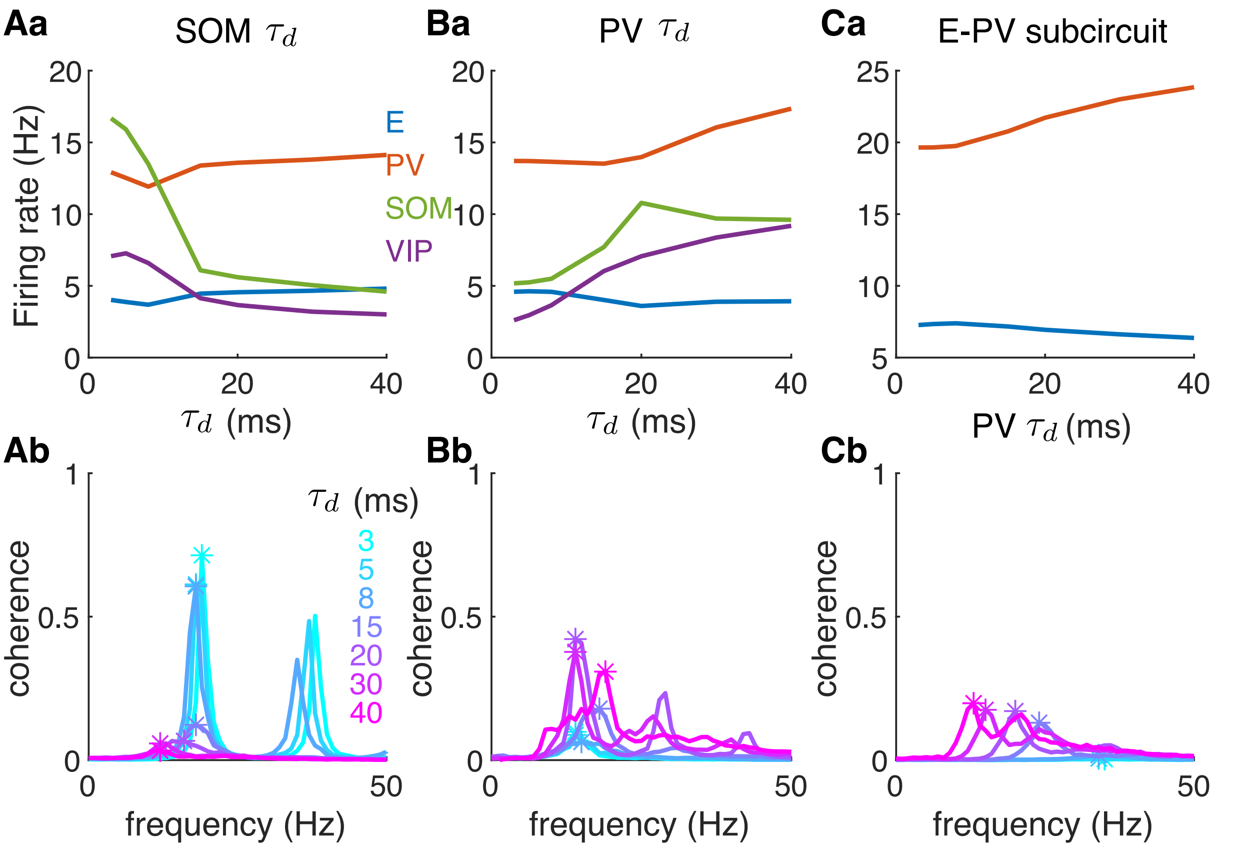

Figure S15: **Impacts of the synaptic time scales on rates and network synchrony. Related to Figure 7B-D.** (A) Varying the decay time constant (τ_d) of SOM synapses. (B) Varying the decay time constant of PV synapses. (C) Same as B in the E-PV subcircuit. Row (a): Average population firing rates of each population as a function of τ_d. Row (b): E population coherence as a function of frequency for networks with different values of τ_d. Other parameters were the same as those in Figure 3. The static input was zero. For reference, τ_d is 8 ms and 20 ms for the PV and SOM synapses, respectively, in the default network in Figure 3.

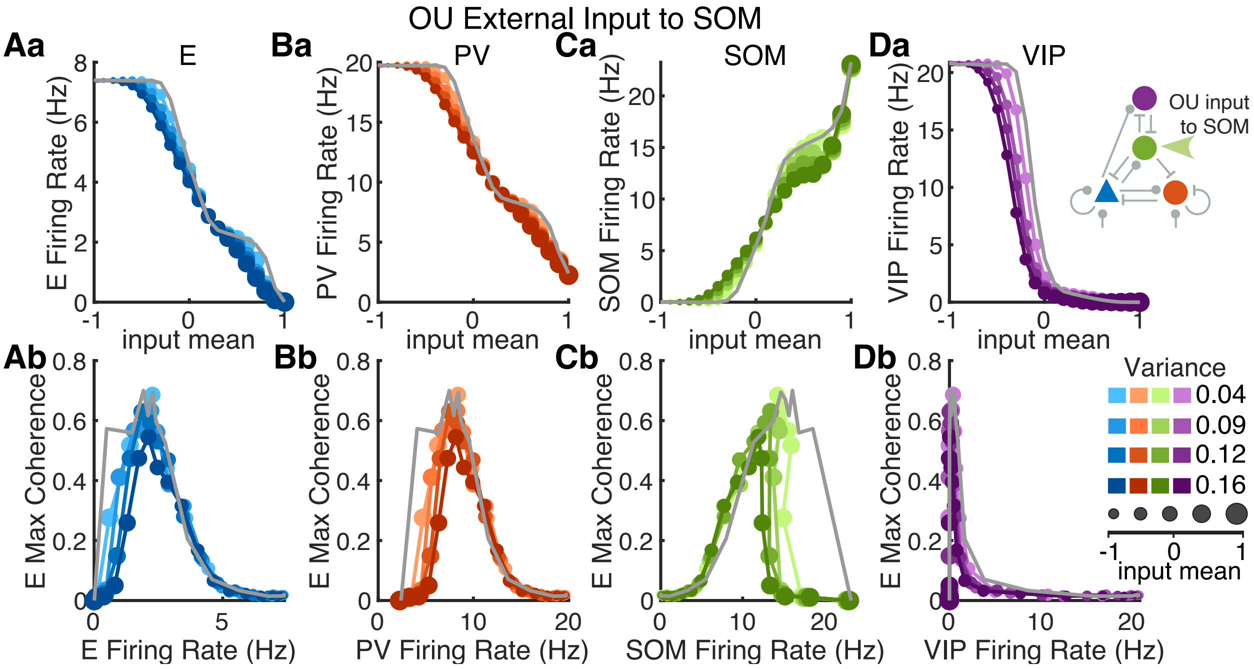

Figure S16: **Weak impacts of dynamic noise input parameters on population rates and coherence. Related to Figure 8.** Independent temporally-varying noise, modeled as an OU process of given mean (dot size) and variance (color shade), is applied to each SOM neuron. Columns show firing rate and coherence of (A) E, (B) PV, (C) SOM, (D) VIP populations. Row (a): Average firing rate of each cell population with respect to the mean value of OU input. Row (b): The maximum coherence of E neurons compared to the population firing rates of each cell type. The grey curves are from the default network with SOM receiving static input.

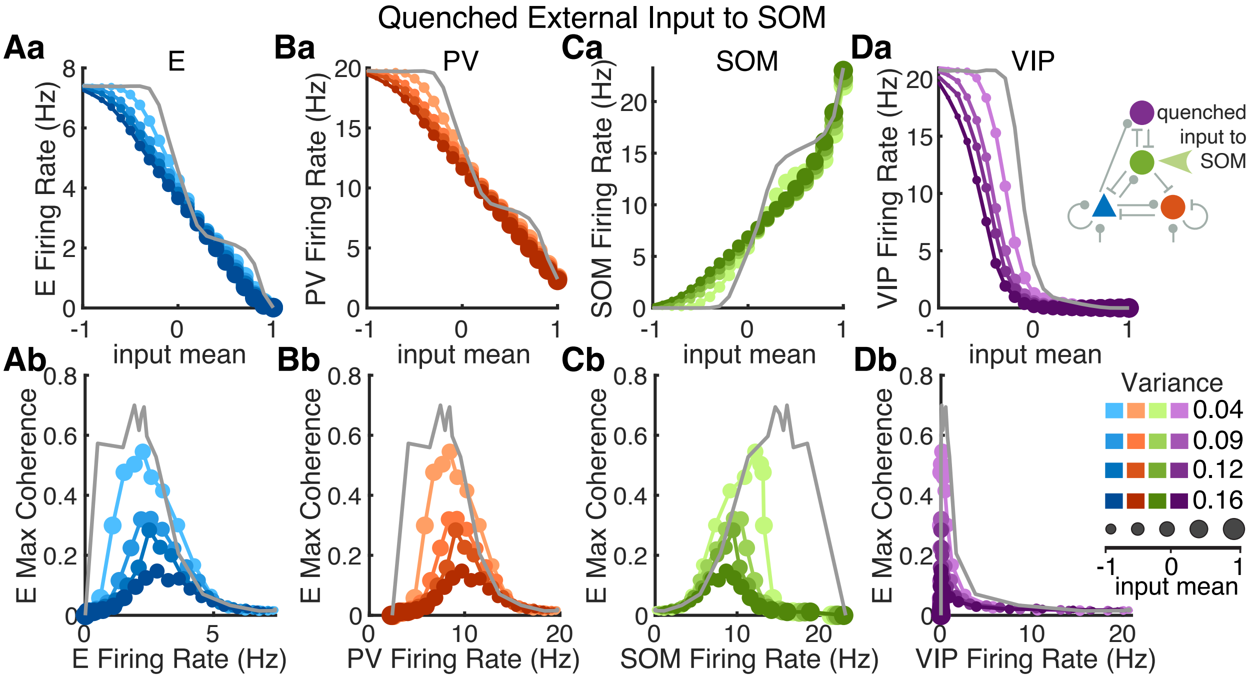

Figure S17: **Impacts of quenched inputs on population rates and coherence. Related to Figure 8.** Quenched input is spatially variable, but temporally invariant. Each SOM neuron receives an input value that is sampled from a Gaussian distribution with given mean (dot size) and variance (color shade). Columns show firing rate and coherence of (A) E, (B) PV, (C) SOM, (D) VIP populations. Row (a): Average firing rate of each cell population with respect to the mean value of the quenched input. Row (b): The maximum coherence of E neurons compared to the population rates of each cell type. The grey curves are from the default network with SOM receiving static input.

Caption for Movie S1. Spiking activities of the spatially dependent spiking neuron network in the subcircuit asynchronous (SA) state (same parameters as in Figure 2A). Each dot indicates that the neuron at spatial position (x, y) fired within one millisecond of the time stamp shown on top. Color of each dot indicates the cell type of the neuron that fired (blue: E; red: PV; green: SOM; purple: VIP).

Caption for Movie S2. Same as Video 1 for the network in the weakly synchronous (WS) state (same parameters as in Figure 2B).

Caption for Movie S3. Same as Video 1 for the network in the strongly synchronous (SS) state (same parameters as in Figure 2C).

REFERENCES AND NOTES

1. T. D. Albright, G. R. Stoner, Contextual influences on visual processing. *Annu. Rev. Neurosci.* **25**, 339–379 (2002).

2. K. D. Harris, A. Thiele, Cortical state and attention. *Nat. Rev. Neurosci.* **12**, 509–523 (2011).

3. D. A. McCormick, D. B. Nestvogel, B. J. He, Neuromodulation of brain state and behavior. *Annu. Rev. Neurosci.* **43**, 391–415 (2020).

4. S. W. Flavell, N. Gogolla, M. Lovett-Barron, M. Zelikowsky, The emergence and influence of internal states. *Neuron* **110**, 2545–2570 (2022).

5. J. S. Isaacson, M. Scanziani, How inhibition shapes cortical activity. *Neuron* **72**, 231–243 (2011).

6. J. A. Cardin, Inhibitory interneurons regulate temporal precision and correlations in cortical circuits. *Trends Neurosci.* **41**, 689–700 (2018).

7. K. A. Ferguson, J. A. Cardin, Mechanisms underlying gain modulation in the cortex. *Nat. Rev. Neurosci.* **21**, 80–92 (2020).

8. Petilla Interneuron Nomenclature Group,G. A. Ascoli, L. Alonso-Nanclares, S. A. Anderson, G. Barrionuevo, R. Benavides-Piccione, A. Burkhalter, G. Buzsáki, B. Cauli, J. DeFelipe, A. Fairén, D. Feldmeyer, G. Fishell, Y. Fregnac, T. F. Freund, D. Gardner, E. P. Gardner, J. H. Goldberg, M. Helmstaedter, S. Hestrin, F. Karube, Z. F. Kisvárday, B. Lambolez, D. A. Lewis, O. Marin, H. Markram, A. Muñoz, A. Packer, C. C. H. Petersen, K. S. Rockland, J. Rossier, B. Rudy, P. Somogyi, J. F. Staiger, G. Tamas, A. M. Thomson, M. Toledo-Rodriguez, Y. Wang, D. C. West, R. Yuste, Petilla terminology: Nomenclature of features of gabaergic interneurons of the cerebral cortex. *Nat. Rev. Neurosci.* **9**, 557–568 (2008).

9. R. Tremblay, S. Lee, B. Rudy, Gabaergic interneurons in the neocortex: From cellular properties to circuits. *Neuron* **91**, 260–292 (2016).

10. G. Chen, M. J. Rasch, R. Wang, X. H. Zhang, Experience-dependent emergence of beta and gamma band oscillations in the primary visual cortex during the critical period. *Sci. Rep.* **5**, 17847 (2015).

11. S. Zhang, M. Xu, T. Kamigaki, J. P. Hoang Do, W.-C. Chang, S. Jenvay, K. Miyamichi, L. Luo, Y. Dan, Long-range and local circuits for top-down modulation of visual cortex processing. *Science* **345**, 660–665 (2014).

12. Y. Fu, J. M. Tucciarone, J. S. Espinosa, N. Sheng, D. P. Darcy, R. A. Nicoll, Z. J. Huang, M. P. Stryker, A cortical circuit for gain control by behavioral state. *Cell* **156**, 1139–1152 (2014).

13. J. M. Pakan, S. C. Lowe, E. Dylda, S. W. Keemink, S. P. Currie, C. A. Coutts, N. L. Rochefort, Behavioral-state modulation of inhibition is context-dependent and cell type specific in mouse visual cortex. *eLife* **5**, e14985 (2016).

14. M. Dipoppa, A. Ranson, M. Krumin, M. Pachitariu, M. Carandini, K. D. Harris, Vision and locomotion shape the interactions between neuron types in mouse visual cortex. *Neuron* **98**, 602–615.e8 (2018).

15. M. Vinck, R. Batista-Brito, U. Knoblich, J. A. Cardin, Arousal and locomotion make distinct contributions to cortical activity patterns and visual encoding. *Neuron* **86**, 740–754 (2015).

16. M. J. McGinley, S. V. David, D. A. McCormick, Cortical membrane potential signature of optimal states for sensory signal detection. *Neuron* **87**, 179–192 (2015).

17. J. Veit, G. Handy, D. P. Mossing, B. Doiron, H. Adesnik, Cortical VIP neurons locally control the gain but globally control the coherence of gamma band rhythms. *Neuron* **111**, 405–417.e5 (2023).

18. G. Chen, Y. Zhang, X. Li, X. Zhao, Q. Ye, Y. Lin, H. W. Tao, M. J. Rasch, X. Zhang, Distinct inhibitory circuits orchestrate cortical *beta* and *gamma* band oscillations. *Neuron* **96**, 1403–1418.e6 (2017).

19. H. J. Jang, H. Chung, J. M. Rowland, B. A. Richards, M. M. Kohl, J. Kwag, Distinct roles of parvalbumin and somatostatin interneurons in gating the synchronization of spike times in the neocortex. *Sci. Adv.* **6**, eaay5333 (2020).

20. K. C. Wood, J. M. Blackwell, M. N. Geffen, Cortical inhibitory interneurons control sensory processing. *Curr. Opin. Neurobiol.* **46**, 200–207 (2017).

21. J. A. Cardin, M. Carlén, K. Meletis, U. Knoblich, F. Zhang, K. Deisseroth, L.-H. Tsai, C. I. Moore, Driving fast-spiking cells induces gamma rhythm and controls sensory responses. *Nature* **459**, 663–667 (2009).

22. V. S. Sohal, F. Zhang, O. Yizhar, K. Deisseroth, Parvalbumin neurons and gamma rhythms enhance cortical circuit performance. *Nature* **459**, 698–702 (2009).

23. G. Buzsáki, X.-J. Wang, Mechanisms of gamma oscillations. *Annu. Rev. Neurosci.* **35**, 203–225 (2012).

24. J. Veit, R. Hakim, M. P. Jadi, T. J. Sejnowski, H. Adesnik, Cortical gamma band synchronization through somatostatin interneurons. *Nat. Neurosci.* **20**, 951–959 (2017).

25. H. Adesnik, W. Bruns, H. Taniguchi, Z. J. Huang, M. Scanziani, A neural circuit for spatial summation in visual cortex. *Nature* **490**, 226, 231 (2012).

26. H.-J. Pi, B. Hangya, D. Kvitsiani, J. I. Sanders, Z. J. Huang, A. Kepecs, Cortical interneurons that specialize in disinhibitory control. *Nature* **503**, 521–524 (2013).

27. A. J. Keller, M. Dipoppa, M. M. Roth, M. S. Caudill, A. Ingrosso, K. D. Miller, M. S. Scanzani, A disinhibitory circuit for contextual modulation in primary visual cortex. *Neuron* **108**, 1181–1193.e8 (2020).

28. K. A. Ferguson, J. Salameh, C. Alba, H. Selwyn, C. Barnes, S. Lohani, J. A. Cardin, VIP interneurons regulate cortical size tuning and visual perception. *Cell Rep.* **42**, 113088 (2023).

29. R. V. Rikhye, M. Yildirim, M. Hu, V. Breton-Provencher, M. Sur, Reliable sensory processing in mouse visual cortex through cooperative interactions between somatostatin and parvalbumin interneurons. *J. Neurosci.* **41**, 8761–8778 (2021).

30. C. Potter, C. Bassi, C. A. Runyan, Simultaneous interneuron labeling reveals population-level interactions among parvalbumin, somatostatin, and pyramidal neurons in cortex. bioRxiv 523298 [Preprint] (2024). https://doi.org/10.1101/2023.01.09.523298.

31. A. Litwin-Kumar, R. Rosenbaum, B. Doiron, Inhibitory stabilization and visual coding in cortical circuits with multiple interneuron subtypes. *J. Neurophysiol.* **115**, 1399–1409 (2016).

32. L. C. Garcia del Molino, G. R. Yang, J. F. Mejias, X.-J. Wang, Paradoxical response reversal of top-down modulation in cortical circuits with three interneuron types. *eLife* **6**, e29742 (2017).

33. A. Palmigiano, F. Fumarola, D. P. Mossing, N. Kraynyukova, H. Adesnik, K. D. Miller, Common rules underlying optogenetic and behavioral modulation of responses in multi-cell-type v1 circuits. bioRxiv 378729 [Preprint] (2020). https://doi.org/10.1101/2020.11.11.378729.

34. F. Waitzmann, Y. K. Wu, J. Gjorgjieva, Top–down modulation in canonical cortical circuits with short-term plasticity. *Proc. Natl. Acad. Sci. U.S.A.* **121**, e2311040121 (2024).

35. L. Beerendonk, J. F. Mejías, S. A. Nuiten, J. W. de Gee, J. J. Fahrenfort, S. van Gaal, A disinhibitory circuit mechanism explains a general principle of peak performance during mid-level arousal. *Proc. Natl. Acad. Sci. U.S.A.* **121**, e2312898121 (2024).

36. D. Vierling-Claassen, J. A. Cardin, C. I. Moore, S. R. Jones, Computational modeling of distinct neocortical oscillations driven by cell-type selective optogenetic drive: Separable resonant circuits controlled by low-threshold spiking and fast-spiking interneurons. *Front. Hum. Neurosci.* **4**, 198 (2010).

37. G. Hahn, A. Kumar, H. Schmidt, T. R. Knösche, G. Deco, Rate and oscillatory switching dynamics of a multilayer visual microcircuit model. *eLife* **11**, e77594 (2022).

38. C. Huang, D. A. Ruff, R. Pyle, R. Rosenbaum, M. R. Cohen, B. Doiron, Circuit models of low-dimensional shared variability in cortical networks. *Neuron* **101**, 337–348.e4 (2019).

39. N. Fourcaud-Trocmé, D. Hansel, C. Van Vreeswijk, N. Brunel, How spike generation mechanisms determine the neuronal response to fluctuating inputs. *J. Neurosci.* **23**, 11628–11640 (2003).

40. L. Campagnola, S. C. Seeman, T. Chartrand, L. Kim, A. Hoggarth, C. Gamlin, S. Ito, J. Trinh, P. Davoudian, C. Radaelli, M. H. Kim, T. Hage, T. Braun, L. Alfiler, J. Andrade, P. Bohn, R. Dalley, A. Henry, S. Kebede, A. Mukora, D. Sandman, G. Williams, R. Larsen, C. Teeter, T. L. Daigle, K. Berry, N. Dotson, R. Enstrom, M. Gorham, M. Hupp, S. Dingman Lee, K. Ngo, P. R. Nicovich, L. Potekhina, S. Ransford, A. Gary, J. Goldy, D. McMillen, T. Pham, M. Tieu, L.'A. Siverts, M. Walker, C. Farrell, M. Schroedter, C. Slaughterbeck, C. Cobb, R. Ellenbogen, R. P. Gwinn, C. D. Keene, A. L. Ko, J. G. Ojemann, D. L. Silbergeld, D. Carey, T. Casper, K. Crichton, M. Clark, N. Dee, L. Ellingwood, J. Gloe, M. Kroll, J. Sulc, H. Tung, K. Wadhwani, K. Brouner, T. Egdorf, M. Maxwell, M. McGraw, C. A. Pom, A. Ruiz, J. Bomben, D. Feng, N. Hejazinia, S. Shi, A. Szafer, W. Wakeman, J. Phillips, A. Bernard, L. Esposito, F. D. D'Orazi, S. Sunkin, K. Smith, B. Tasic, A. Arkhipov, S. Sorensen, E. Lein, C. Koch, G. Murphy, H. Zeng, T. Jarsky, Local connectivity and synaptic dynamics in mouse and human neocortex. *Science* **375**, eabj5861 (2022).

41. C. K. Pfeffer, M. Xue, M. He, Z. J. Huang, M. Scanziani, Inhibition of inhibition in visual cortex: the logic of connections between molecularly distinct interneurons. *Nat. Neurosci.* **16**, 1068–1076 (2013).

42. N. Mosheiff, B. Ermentrout, C. Huang, Chaotic dynamics in spatially distributed neuronal networks generate population-wide shared variability. *PLoS Comput. Biol.* **19**, e1010843 (2023).

43. H. K. Kato, S. K. Asinof, J. S. Isaacson, Network-level control of frequency tuning in auditory cortex. *Neuron* **95**, 412–423.e4 (2017).

44. M. M. Karnani, J. Jackson, I. Ayzenshtat, J. Tucciarone, K. Manoocheri, W. G. Snider, R. Yuste, Cooperative subnetworks of molecularly similar interneurons in mouse neocortex. *Neuron* **90**, 86–100 (2016).

45. Y. Kim, G. R. Yang, K. Pradhan, K. U. Venkataraju, M. Bota, L. C. García del Molino, G. Fitzgerald, K. Ram, M. He, J. M. Levine, P. Mitra, Z. J. Huang, X.-J. Wang, P. Osten, Brain-wide maps reveal stereotyped cell-type-based cortical architecture and subcortical sexual dimorphism. *Cell* **171**, 456–469.e22 (2017).

46. C. F. Khoury, N. G. Fala, C. A. Runyan, The spatial scale of somatostatin subnetworks increases from sensory to association cortex. *Cell Rep.* **40**, 111319 (2022).

47. M. J. Richardson, Firing-rate response of linear and nonlinear integrate-and-fire neurons to modulated current-based and conductance-based synaptic drive. *Phys. Rev. E Stat. Nonlin. Soft Mat. Phys.* **76**, 021919 (2007).

48. R. Rosenbaum, J. Trousdale, K. Josic, Pooling and correlated neural activity. *Front. Comput. Neurosci.* **4**, 1209 (2010).

49. A. Renart, J. de la Rocha, P. Bartho, L. Hollender, N. Parga, A. Reyes, K. D. Harris, The asynchronous state in cortical circuits. *Science* **327**, 587–590 (2010).

50. C. Van Vreeswijk, H. Sompolinsky, Chaos in neuronal networks with balanced excitatory and inhibitory activity. *Science* **274**, 1724–1726 (1996).

51. J. De La Rocha, B. Doiron, E. Shea-Brown, K. Josić, A. Reyes, Correlation between neural spike trains increases with firing rate. *Nature* **448**, 802–806 (2007).

52. R. Gast, S. A. Solla, A. Kennedy, Neural heterogeneity controls computations in spiking neural networks. *Proc. Natl. Acad. Sci. U.S.A.* **121**, e2311885121 (2024).

53. S. Rich, H. M. Chameh, J. Lefebvre, T. A. Valiante, Loss of neuronal heterogeneity in epileptogenic human tissue impairs network resilience to sudden changes in synchrony. *Cell Rep.* **39**, 110863 (2022).

54. J. F. Mejias, A. Longtin, Differential effects of excitatory and inhibitory heterogeneity on the gain and asynchronous state of sparse cortical networks. *Front. Comput. Neurosci.* **8**, 107 (2014).

55. J. Mejias, A. Longtin, Optimal heterogeneity for coding in spiking neural networks. *Phys. Rev. Lett.* **108**, 228102 (2012).

56. M. R. Cohen, J. H. R. Maunsell, Attention improves performance primarily by reducing interneuronal correlations. *Nat. Neurosci.* **12**, 1594–1600 (2009).

57. J. Reimer, E. Froudarakis, C. R. Cadwell, D. Yatsenko, G. H. Denfield, A. S. Tolias, Pupil fluctuations track fast switching of cortical states during quiet wakefulness. *Neuron* **84**, 355–362 (2014).

58. A. Kohn, R. Coen-Cagli, I. Kanitscheider, A. Pouget, Correlations and neuronal population information. *Annu. Rev. Neurosci.* **39**, 237–256 (2016).

59. H. Bos, C. Miehl, A.-M. Oswald, B. Doiron, Untangling stability and gain modulation in cortical circuits with multiple interneuron classes. *eLife* **13**, RP99808 (2025).

60. H. Xu, H.-Y. Jeong, R. Tremblay, B. Rudy, Neocortical somatostatin-expressing gabaergic interneurons disinhibit the thalamorecipient layer 4. *Neuron* **77**, 155–167 (2013).

61. M. Garrett, S. Manavi, K. Roll, D. R. Ollerenshaw, P. A. Groblewski, N. D. Ponvert, J. T. Kiggins, L. Casal, K. Mace, A. Williford, A. Leon, X. Jia, P. Ledochowitsch, M. A. Buice, W. Wakeman, S. Mihalas, S. R. Olsen, Experience shapes activity dynamics and stimulus coding of VIP inhibitory cells. *eLife* **9**, e50340 (2020).

62. F. Najafi, S. Russo, J. Lecoq, Unexpected events modulate context signaling in VIP and excitatory cells of the visual cortex. bioRxiv 594366 [Preprint] (2024). https://doi.org/10.1101/2024.05.15.594366.

63. J. H. Lee, C. Koch, S. Mihalas, A computational analysis of the function of three inhibitory cell types in contextual visual processing. *Front. Comput. Neurosci.* **11**, 28 (2017).

64. J. H. Lee, S. Mihalas, Visual processing mode switching regulated by VIP cells. *Sci. Rep.* **7**, 1843 (2017).

65. J. W. Domhof, P. H. Tiesinga, Flexible frequency switching in adult mouse visual cortex is mediated by competition between parvalbumin and somatostatin expressing interneurons. *Neural Comput.* **33**, 926–966 (2021).

66. Y. N. Billeh, B. Cai, S. L. Gratiy, K. Dai, R. Iyer, N. W. Gouwens, R. Abbasi-Asl, X. Jia, J. H. Siegle, S. R. Olsen, C. Koch, S. Mihalas, A. Arkhipov, Systematic integration of structural and functional data into multi-scale models of mouse primary visual cortex. *Neuron* **106**, 388–403.e18 (2020).

67. N. Wagatsuma, S. Nobukawa, T. Fukai, A microcircuit model involving parvalbumin, somatostatin, and vasoactive intestinal polypeptide inhibitory interneurons for the modulation of neuronal oscillation during visual processing. *Cereb. Cortex* **33**, 4459–4477 (2023).

68. B. Lee, D. Shin, S. P. Gross, K.-H. Cho, Combined positive and negative feedback allows modulation of neuronal oscillation frequency during sensory processing. *Cell Rep.* **25**, 1548–1560.e3 (2018).

69. G. R. Yang, J. D. Murray, X.-J. Wang, A dendritic disinhibitory circuit mechanism for pathway-specific gating. *Nat. Commun.* **7**, 12815 (2016).

70. L. Hertäg, H. Sprekeler, Amplifying the redistribution of somato-dendritic inhibition by the interplay of three interneuron types. *PLOS Comput. Biol.* **15**, e1006999 (2019).

71. L. Hertäg, H. Sprekeler, Learning prediction error neurons in a canonical interneuron circuit. *eLife* **9**, e57541 (2020).

72. S. Akella, P. Ledochowitsch, J. H. Siegle, H. Belski, D. Denman, M. A. Buice, S. Durand, C. Koch, S. R. Olsen, X. Jia, Deciphering neuronal variability across states reveals dynamic sensory encoding. *Nat. Commun.* **16**, 1768 (2025).

73. T. C. Potjans, M. Diesmann, The cell-type specific cortical microcircuit: relating structure and activity in a full-scale spiking network model. *Cereb. Cortex* **24**, 785–806 (2014).

74. H. Bos, M. Diesmann, M. Helias, Identifying anatomical origins of coexisting oscillations in the cortical microcircuit. *PLoS Comput. Biol.* **12**, e1005132 (2016).

75. M. V. Tsodyks, W. E. Skaggs, T. J. Sejnowski, B. L. McNaughton, Paradoxical effects of external modulation of inhibitory interneurons. *J. Neurosci.* **17**, 4382–4388 (1997).

76. H. Ozeki, I. M. Finn, E. S. Schaffer, K. D. Miller, D. Ferster, Inhibitory stabilization of the cortical network underlies visual surround suppression. *Neuron* **62**, 578–592 (2009).

77. A. Sanzeni, B. Akitake, H. C. Goldbach, C. E. Leedy, N. Brunel, M. H. Histed, Inhibition stabilization is a widespread property of cortical networks. *eLife* **9**, e54875 (2020).
